# Early total care or damage control orthopaedics for major fractures ? Results of propensity score matching for early definitive versus early temporary fixation based on data from the trauma registry of the German Trauma Society (TraumaRegister DGU^®^)

**DOI:** 10.1007/s00068-022-02215-3

**Published:** 2023-01-20

**Authors:** Falk von Lübken, Sascha Prause, Patricia Lang, Benedikt Dieter Friemert, Rolf Lefering, Gerhard Achatz

**Affiliations:** 1 Department of Trauma Surgery and Orthopaedics, Reconstructive and Septic Surgery, and Sports Traumatology, German Armed Forces Hospital of Ulm, Oberer Eselsberg 40, 89081 Ulm, Germany; 2 Department of Anaesthesiology, Intensive Care Medicine, Emergency Medicine, and Pain Therapy, German Armed Forces Hospital of Ulm, Ulm, Germany; 3 Centre for Integrated Rehabilitation, Rehabilitation Hospital of Ulm, Ulm, Germany; 4 Institute for Research in Operative Medicine, Witten/Herdecke University, Cologne, Germany

**Keywords:** Damage control orthopaedics, Early total care, Polytrauma, Trauma surgery, Propensity score

## Abstract

**Purpose:**

Damage control orthopaedics (DCO) und early total care (ETC) are well-established strategies for managing severely injured patients. There is no definitive evidence of the superiority of DCO over ETC in polytrauma patients. We conducted this study to assess the probability of a polytraumatised patient undergoing DCO. In addition, the effect of DCO on complications and mortality was investigated.

**Methods:**

We analysed data from 12,569 patients with severe trauma (Injury Severity Score ≥ 16) who were enrolled in the trauma registry of the German Trauma Society (TraumaRegister DGU^®^) from 2009 to 2016 and had undergone surgery for extremity or pelvic fractures. These patients were allocated to a DCO or an ETC group. We used the propensity score to identify factors supporting the use of DCO. For a comparison of mortality rates, the groups were stratified and matched on the propensity score.

**Results:**

We identified relevant differences between DCO and ETC. DCO was considerably more often associated with packed red blood cell (pRBC) transfusions (33.9% vs. 13.4%), catecholamine therapy (14.1% vs. 6.8%), lower extremity injuries (72.4% vs. 53.5%), unstable pelvic fractures (41.0% vs. 25.9%), penetrating injuries (2.8% vs. 1.5%), and shock (20.5% vs. 10.8%) and unconsciousness (23.7% vs. 16.3%) on admission. Based on the propensity score, patients with penetrating trauma, pRBC transfusions, unstable pelvic fractures, and lower extremity injuries were more likely to undergo DCO. A benefit of DCO such as reduced complications or reduced mortality was not detected.

**Conclusion:**

We could identify some parameters of polytrauma patients used in the trauma registry (Traumaregister DGU^®^), which led more likely to a DCO therapy. The propensity score did not demonstrate the superiority of DCO over ETC in terms of outcome or complications. It did not appear to adequately adjust for the variables used here. Definitive evidence for or against the use of DCO remains unavailable.

## Introduction

The term ‘damage control’ refers to a treatment strategy that aims to keep the impact of initial surgical treatment to the minimum level necessary in severely injured patients [24]. Rotondo et al. proposed the damage control approach in 1993 in order to address the large incidence of penetrating injuries in the United States [49]. The concept of damage control surgery focuses on ensuring the immediate survival of injured patients by reducing the initial treatment measures, which are usually surgical in nature, and on rapidly stabilising patients in the medical intensive care setting. Based on this concept, damage control orthopaedics (DCO) describes a similar approach for the management of injuries to the musculoskeletal system. This applies in particular to the stabilisation of pelvic and extremity fractures, usually with the use of external fixation systems, until definitive care can be provided [17]. By contrast, early total care (ETC) refers to the primary definitive treatment of such injuries, for example with primary intramedullary nailing or plate fixation.

Since the 1980s, clinical research has addressed the question of whether it is more appropriate for polytrauma patients to undergo DCO or ETC. Behrman et al. and Harvin et al. showed in their retrospective studies that the early fixation of femur fractures was associated with a reduction in pulmonary complications and health care costs [2, 21]. They investigated, however, only patients who underwent intramedullary nailing, which is a typical ETC approach in the group of patients who received early definitive care. Charash et al. carried out a retrospective study to determine whether early intramedullary nailing of femoral fractures in polytrauma patients with an Injury Severity Score (ISS) ≥ 18 led to a higher rate of pulmonary complications when thoracic trauma was present or absent [7]. They reported that the risk of pulmonary complications, the length of hospital stay, and the length of stay in the intensive care unit (ICU) tended to be greater in patients with delayed intramedullary nailing of femur fractures regardless of whether or not they had sustained thoracic trauma. The differences between the patient groups, however, were not significant.

In 2016, Liu et al. conducted a meta-analysis and they too found no significant differences in the rate of pulmonary complications, multiple organ failure, and mortality between early and delayed intramedullary nailing of femur fractures in patients with concomitant severe thoracic trauma [33]. Early definitive care thus does not appear to have any disadvantages in comparison to delayed definitive treatment. The aforementioned studies, however, investigated the timing of surgery and did not directly compare DCO and ETC. Pape et al. addressed this issue in 2007 and conducted a prospective randomised clinical study on patients with multiple injuries [40]. They compared stable patients and patients at risk of complications (borderline patients). ETC for femur fractures in stable patients was associated with shorter ventilation times and a lower incidence of pulmonary failure and sepsis than DCO. By contrast, borderline patients who underwent ETC had a significantly higher rate of pulmonary complications than those who received DCO. Unstable patients were excluded. Tuttle et al. compared multiply injured patients who underwent either DCO (*n = *55) or ETC (*n = *42) for femoral shaft fractures and found no significant differences between the groups for adult respiratory distress syndrome (ARDS), multiple organ failure, ICU length of stay, and hospital length of stay [54]. DCO patients, however, had a significantly shorter operative time and less estimated blood loss from their initial surgical procedure. According to Giannoudis, ETC is indicated in patients with stable haemodynamics, stable oxygen saturation, a lactate level < 2 mmol/L, no coagulation disturbances, a normal temperature, urinary output > 1 mL/kg/hour, and no requirement for inotropic support [13]. Although this description of patients who are suitable for ETC is different from the definition of stable patients by Pape et al. [40], they point in the same direction. In other studies investigating different treatment strategies, the role of DCO in the management of polytrauma patients is controversial [1, 22, 23, 36, 37, 45, 52, 53]. Rixen et al. noted, for example, that there was no data that met the requirements of evidence-based medicine and demonstrated the superiority of damage control orthopaedics over early total care [47].

As described above, the decision to use the DCO approach in the management of patients depends on a variety of factors including patient-related variables such as age and gender as well as injury-related and treatment-related variables. Examples of injury-related variables are injury severity and the mechanism of injury. Treatment-related variables are the level of the treating trauma centre and the time of treatment. Additional variables may be taken into consideration as well [31].

These variables provide the basis for propensity score analyses. The propensity score is defined as the probability that a patient receives a given treatment [6, 29]. In our study, this treatment is the management of an extremity injury using the DCO approach. The propensity score is also used to estimate the effects of treatment according to DCO principles on outcome, which is of particular interest here. The propensity score is a useful alternative method for analysing non-randomised studies and registry data [29–31].

In the study presented here, we hypothesised that the management of polytrauma patients on the basis of DCO principles is superior to an ETC approach in terms of outcome and complications. By this we agree with Scalea, who writes in 2002 that polytrauma patients benefit from initial surgical care that addresses only the haemorrhage, and are then further stabilised (in intensive care) [51]. Final stabilisation could then be done later. He writes this based on his impressions from the drug wars of the 1980s and 1990s, where patients with multiple injuries died despite successful interventions.

The main objectives of our study were:

1. to assess what variables are associated with the use of DCO on the basis of data from the trauma registry of the German Trauma Society (TraumaRegister DGU^®^) and on the basis of the propensity scores that we calculated using these registry data and
2. to evaluate the effect of DCO on complications and outcome on the basis of propensity scores.

## Material and methods

### TraumaRegister DGU^®^

The TraumaRegister DGU^®^ of the German Trauma Society (Deutsche Gesellschaft für Unfallchirurgie, DGU) was founded in 1993. The aim of this multi-centre database is the pseudonymised and standardised documentation of severely injured patients. Data are collected prospectively in four consecutive time phases from the site of the accident until discharge from hospital: (A) prehospital phase, (B) emergency/resuscitation room and initial surgery, (C) intensive care unit, and (D) discharge.

Documentation includes detailed information on demographics, injury patterns, comorbidities, prehospital and inhospital management, the course on the intensive care unit, relevant laboratory findings including data on transfusion, and outcome. Included are patients who are admitted to hospital via the resuscitation room and subsequently receive intensive or intermediate care and patients who arrive at hospital with vital signs and die before admission to the intensive care unit. The infrastructure for documentation, data management, and data analysis is provided by the Academy for Trauma Surgery (Akademie der Unfallchirurgie GmbH, AUC), which is affiliated to the German Trauma Society. Scientific leadership is provided by the Committee on Emergency Medicine, Intensive Care and Trauma Management (Sektion NIS) of the German Trauma Society. Participating hospitals submit their pseudonymised data to a central database via a web-based application. Scientific data analysis is approved according to a peer review process described in the publication guideline of TraumaRegister DGU^®^. The participating hospitals are primarily located in Germany (90%), but a growing number of hospitals in other countries contribute data as well (i.e. Austria, Belgium, Finland, Luxembourg, Slovenia, Switzerland, the Netherlands, and the United Arab Emirates). Currently, approximately 30,000 cases from more than 650 hospitals are entered into the database per year. Participation in the TraumaRegister DGU^®^ is voluntary. For hospitals associated with TraumaNetzwerk DGU^®^, however, the entry of at least a basic data set is obligatory for reasons of quality assurance.

The study presented here was performed in accordance with the publication guideline of the TraumaRegister DGU^®^ and is registered as TR-DGU Project ID 2016-011.

### Patients

**Fig. 1:**
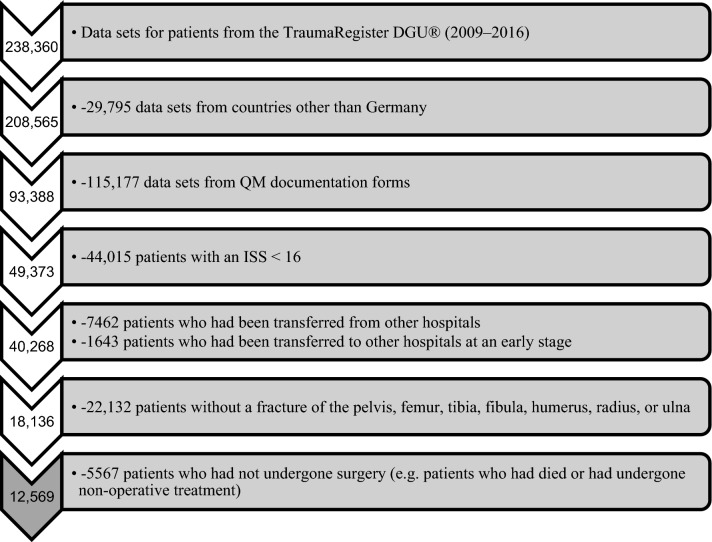
Patient identification process

We analysed data from 12,569 patients who were enrolled in the trauma registry from 2009 to 2016. Figure 1 shows how these patients were identified from the 238,360 data sets that were available in the TraumaRegister DGU^®^ from 2009 to 2016. Included were patients with an Injury Severity Score (ISS) ≥ 16 who had been admitted to hospital via the resuscitation room, had undergone surgery, and had then been transferred to the intensive care unit. Only primary admissions to local, regional or supraregional trauma centres in Germany were included. The patients who were enrolled in the study had been treated in a total of 176 hospitals. Only data from standard documentation forms were used since the QM documentation form does not include relevant parameters. Excluded were patients without a fracture of the pelvis, femur, tibia, fibula, humerus, radius, or ulna. In the last line of Fig. 1, 5567 patients were excluded, because they had not undergone surgery. 1679 of them died during their hospital stay, 1132 of them within the first 24 h after arrival. The remaining 3888 patients had not undergone surgery (e.g. conservative therapy) or the documentation of the surgical procederes were not available.

### Patient groups

The 12,569 patients who were included in the study were divided into two groups. The damage control orthopaedics (DCO) group consisted of patients who were managed on the basis of damage control principles and patients who were treated with external fixation of at least one long bone fracture and/or a pelvis fracture. Patients in the DCO group were compared with all other patients who underwent surgery, so the fractures of those patients were treated only with a definitive technique such as a medullary nail or a plate without getting an external fixator first. These patients formed the early total care (ETC) group.

### Statistical analysis

Descriptive statistics were expressed as percentages and numbers for categorical variables and as means and standard deviations (SDs) for metric variables. Median and interquartile ranges (IQRs) were reported for highly skewed data. The chi-square test was used for comparing frequencies and the Mann–Whitney *U* test for metric data (regardless of data distribution). Because of the large number of patients and the wide variety of comparisons, statistical significance should be interpreted with caution.

The propensity score is defined as the probability that a given intervention or treatment is provided [31]. In our study, we calculated the probability that a patient underwent DCO. Treatment according to DCO principles was the dependent variable and the transfusion of packed red blood cells (pRBCs), patient age, etc. were independent or predictor variables.

Propensity scores were obtained using logistic regression. This approach allowed adjusted effects of possible predictors to be calculated and presented using odds ratios (ORs).

The goodness-of-fit of the model was assessed using coefficients of determination, i.e. the Cox and Snell pseudo-*R*^2^ and the Nagelkerke pseudo-*R*^2^. The value of coefficients of determination is between 0 and 1. The higher the value, the better the goodness-of-fit of the model.

Propensity scores were used to form deciles of probability for comparisons of outcome (mortality). In addition, propensity scores were used for exact matching on the basis of rounded percentages. A patient from the DCO group was matched with a patient from the ETC group with the same propensity score.

Data were analysed using the SPSS software package (version 24, IBM Inc., Armonk, NY, United States).

We divided the variables in the TraumaRegister DGU^®^ into groups of variables. Propensity scores were calculated on the basis of the variables listed in Table 1.

**Table 1:** Independent variables for calculating propensity scores

Demographics	Mechanism of trauma	Injuries	Level of trauma centre	Time of treatment	Treatment
Gender	Blunt or penetrating	Head injuries	Supraregional trauma centre	On-call setting	Prehospital intubation
Age group					
1–15 years	Fall from height	Thoracic trauma Abdominal trauma Upper extremity injury	Regional trauma centre Local trauma centre		Prehospital fluid administration Prehospital catecholamine therapy Transfusion of pRBCs
16–59 years		Lower extremity injury Pelvic injuries			
60–69 years		Most severe injury			
≥ 70 years		Number of injuries			

### Ethics committee

The Ethics Committee of the University of Ulm informed us on 12 June 2017 that our study did not need ethical approval.

## Results

### Baseline characteristics

A total of 12,569 patients were included in the study. Of these, 8199 (65%) were allocated to the DCO group and 4370 (35%) to the ETC group. Tables 2 and 3 provide an overview of baseline characteristics of the patients in the DCO and ETC groups (i.e. demographics, mechanism of trauma, injuries, prehospital physiology and treatment, inhospital care, complications and outcome).

**Table 2:** Baseline characteristics of patients in the DCO and ETC groups–injuries and prehospital care

Parameter	Damage control *n = *8199	Early total care *n = *4370	*p* value
Demographics			
Male gender	70.0% (5786)	69.0% (3002)	0.027
Age (in years)	44.8 (SD 19.7)	47.2 (SD 21.2)	< 0.001
Mechanism of trauma			
Penetrating trauma	2.8% (222)	1.5% (64)	< 0.001
Cause of injury			< 0.001
Road traffic accident (car/truck)	31.5% (2549)	29.2% (1258)	
Road traffic accident (motorcycle)	20.6% (1662)	16.4% (708)	
Road traffic accident (bicycle)	5.0% (402)	5.6% (241)	
Road traffic accident (pedestrian)	11.8% (951)	9.8% (424)	
Fall from > 3 m	21.6% (1746)	23.6% (1017)	
Fall from < 3 m	3.3% (263)	9.7% (416)	
Injuries			
Injury Severity Score (ISS)	30.5 (SD 12.3)	25.9 (9.6)	< 0.001
Number of injuries	7.5 (SD 3.5)	6.3 (SD 1.9)	< 0.001
Head injury (AIS 3 +)	34.9% (2863)	35.2% (1540)	0.72
Thoracic trauma (AIS 3 +)	63.7% (5224)	60.5% (2642)	< 0.001
Abdominal trauma (AIS 3 +)	23.5% (1923)	16.1% (704)	< 0.001
Injury to an upper extremity (AIS 2 +)	53.5% (4383)	58.7% (2566)	< 0.001
Injury to a lower extremity (without pelvis, AIS 2 +)	72.4% (5934)	53.5% (2339)	< 0.001
Stable pelvic injury (AIS 2)	10.1% (824)	12.6% (551)	< 0.001
Unstable pelvic injury (AIS 3–5)	41.0% (3360)	25.9% (1132)	
Femur fracture	41.0% (3363)	27.8% (1217)	< 0.001
Multiple extremity fractures	59.3% (4866)	37,3% (1628)	< 0.001
Prehospital physiology and treatment			
Systolic blood pressure (mmHg)	118 (32)	126 (30)	< 0.001
Shock (BP ≤ 90 mmHg)	21.0% (1535)	12.1% (478)	< 0.001
Shock on arrival	20.5% (1577)	10.8% (441)	< 0.001
Glasgow Coma Scale (score)	11.7 (4.4)	12.6 (3.8)	< 0.001
Unconsciousness (GCS < 9)	23.7% (1831)	16.3% (669)	< 0.001
Fluid administration > 1000 mL	36.9% (3029)	22.3% (976)	< 0.001
Intubation	50.8% (4118)	34.0% (1462)	< 0.001
Catecholamine therapy	14.1% (1140)	6.8% (294)	< 0.001
Care			
Receiving hospital/trauma centre			< 0.001
Level 1 (supraregional)	88.8% (7277)	84.1% (3676)	
Level 2 (regional)	10.2% (833)	13.5% (590)	
Level 3 (local)	1.1% (89)	2.4% (104)	
Prehospital rescue time (in minutes)	70.9 (SD 27.9)	68.7 (SD 27.7)	< 0.001
Admission in the on-call setting (4:00 p.m. to 7:00 a.m.)	67.9% (5517)	64.6% (2805)	< 0.001

**Table 3:** Baseline characteristics of patients in the DCO and ETC groups–inhospital care and outcome

Parameter	DCO	ETC	*p* value
Inhospital treatment			
Transfusion of pRBCs in the resuscitation room	33.9% (2774)	13.4% (586)	< 0.001
Massive transfusions (≥ 10 units of pRBCs)	8.6% (703)	1.8% (80)	< 0.001
Intensive care	97.1% (7958)	96.2% (4202)	0.006
Number of surgical procedures	7.1 (SD 6.6)	3.7 (SD 3.4)	< 0.001
Outcome			
Duration of ventilation^a^ (in days)	2 (0–10)	1 (0–5)	< 0.001
Length of ICU stay^a^ (in days)	8 (3–20)	3 (2–13)	< 0.001
Length of hospital stay^a^ (in days)	26 (17–40)	20 (14–29)	< 0.001
Sepsis	10.6% (804)	6.1% (245)	< 0.001
Multiple organ failure	36.4% (2836)	21.3% (876)	< 0.001
Mortality within the first 24 h	3.9% (322)	0.8% (34)	< 0.001
Pulmonary failure	28.1% (2189)	17.3% (711)	< 0.001
Inhospital mortality	10.8% (887)	3.8% (167)	< 0.001

^a^Expressed as medians and interquartile ranges (IQRs)

A comparison of the treatment groups showed a much higher percentage of penetrating trauma and a much lower percentage of falls from a height of less than 3 m in the DCO group. The percentages of patients with lower extremity injuries and unstable pelvic injuries were also considerably higher in the DCO group. The same applies to the presence of shock (defined as a blood pressure ≤ 90 mmHg) and unconsciousness, prehospital catecholamine therapy, pRBC transfusions in the resuscitation room, and the total number of surgical procedures. A comparison of patient outcomes showed that the patients in the DCO group had longer ICU lengths of stay, had sepsis more frequently, and had considerably higher mortality rates than the ETC group.

### Propensity score

We were able to calculate propensity scores for 12,033 patients (96%) with complete data. The following variables were found to have no major effect (*p > *0.10): gender, abdominal trauma, upper extremity injury, and coagulopathy. In accordance with Pape et al. [39], coagulopathy was defined as a prolonged partial thromboplastin time (PTT) of 50 s or more or an International Normalised Ratio (INR) of 1.4 or higher.

**Fig. 2:**
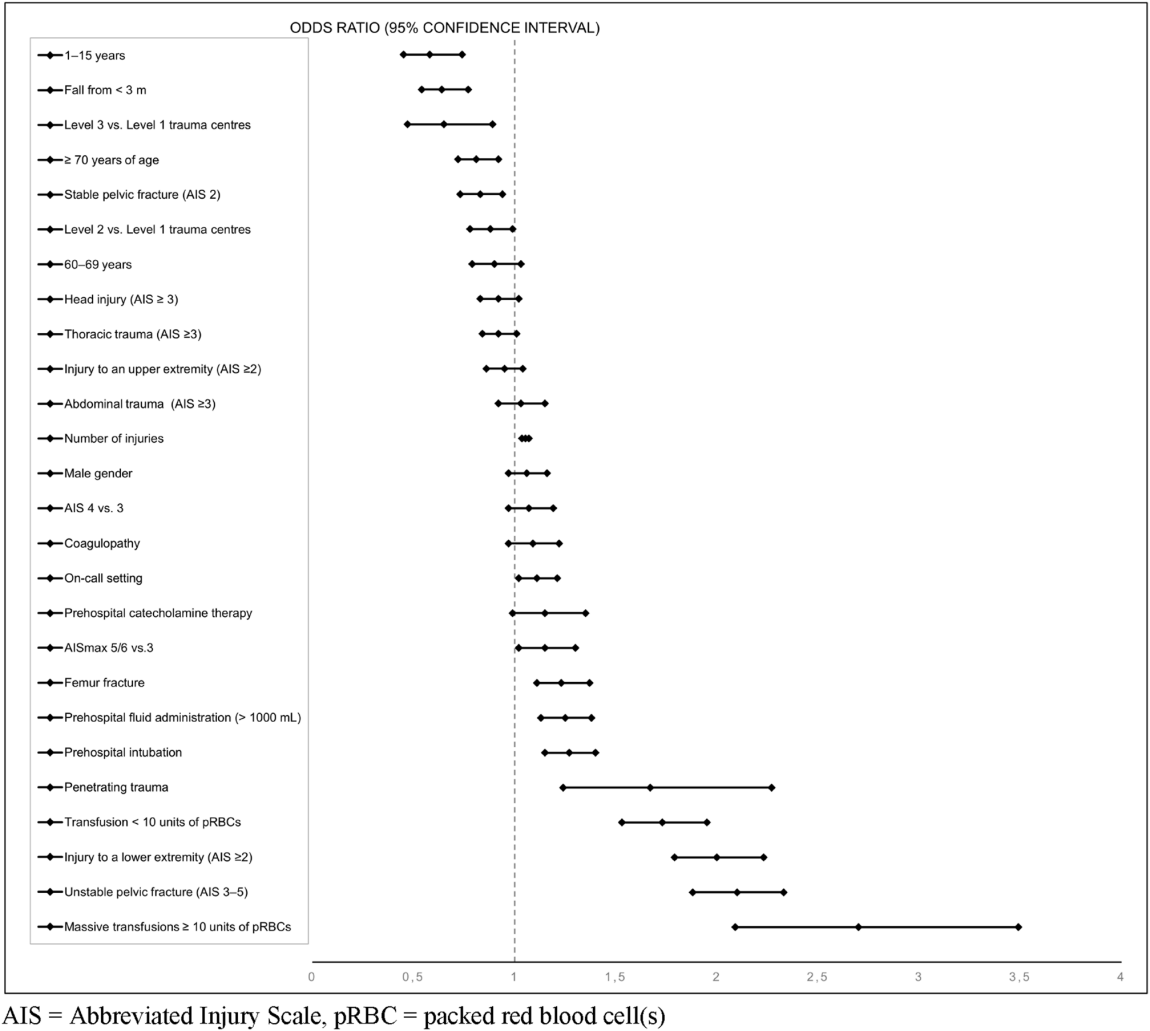
Forest plot displaying the results of the logistic regression. *AIS* Abbreviated Injury Scale, *pRBC* packed red blood cell(s)

Based on odds ratios, the variables that were most strongly associated with the DCO approach were lower extremity fractures, unstable pelvic fractures, and transfusion requirements prior to admission to the ICU, the presence of penetrating trauma, and the number of injuries. Odds ratios suggested that ETC was most commonly used in young patients (under 16 years of age) and in patients whose injuries resulted from a fall from a low height. Table 4 provides an overview of these data. A forest plot graphically displaying the logistic regression is shown in Fig. 2. It demonstrates, for example, that there were no relevant differences between treatment provided in supraregional and regional trauma centres since the confidence intervals for the odds ratios were close to 1. Most parameters were very close to 1. The closer the values are to 1, the more difficult it is to identify an independent variable. In our study, an odds ratio of 1 meant that the DCO approach and the ETC approach were equally likely to be used in the management of patients.

**Table 4:** Logistic regression analysis for propensity score calculation; dependent variable: damage control orthopaedics. Included were 12,033 patients with complete data (96%)

	Regression coefficient	Odds ratio (OR)	95% confidence interval for OR	Significance
Age (reference: 16–59 years)				< 0.001
1–15 years	− 0.55	0.58	0.45–0.74	< 0.001
60–69 years	− 0.11	0.90	0.79–1.03	0.123
70 years and older	− 0.21	0.81	0.72–0.92	0.001
Male gender	0.06	1.06	0.97–1.16	0.193
Penetrating trauma	0.51	1.67	1.24–2.27	0.001
Head injury (AIS ≥ 3)	− 0.08	0.92	0.83–1.02	0.097
Thoracic trauma (AIS ≥ 3)	− 0.08	0.92	0.84–1.01	0.083
Abdominal trauma (AIS ≥ 3)	0.03	1.03	0.92–1.15	0.580
Injury to an upper extremity (AIS ≥ 2)	− 0.06	0.95	0.86–1.04	0.239
Injury to a lower extremity (AIS ≥ 2)	0.69	2.00	1.79–2.23	< 0.001
Femur fracture	0.21	1.23	1.11–1.37	< 0.001
Stable pelvic fracture (AIS 2)	− 0.19	0.83	0.73–0.94	0.005
Unstable pelvic fracture (AIS 3–5)	0.74	2.10	1.88–2.33	< 0.001
Most severe injury (reference: AIS 3)				0.077
AIS 4	0.07	1.07	0.97–1.19	0.153
AIS 5/6	0.14	1.15	1.02–1.30	0.027
Number of injuries (per injury)	0.052	1.053	1.036–1.070	< 0.001
Prehospital intubation	0.24	1.27	1.15–1.40	< 0.001
Prehospital catecholamine therapy	0.14	1.15	0.99–1.35	0.072
Prehospital fluid administration (> 1000 mL)	0.22	1.25	1.13–1.38	< 0.001
Coagulopathy	0.08	1.09	0.97–1.22	0.166
Transfusion of pRBC units (no reference)				< 0.001
1–9 units	0.55	1.73	1.53–1.95	< 0.001
Massive transfusions (≥ 10 units)	0.99	2.70	2.09–3.49	< 0.009
Trauma centre (reference: Level 1)				
Level 2	− 0.13	0.88	0.78–0.99	0.045
Level 3	− 0.43	0.65	0.47–0.89	0.008
Fall from less than 3 m	− 0.44	0.64	0.54–0.77	< 0.001
On-call setting	0.10	1.11	1.02–1.21	0.016
Constant	− 0.77			< 0.001

The Nagelkerke *R*^2^ = 0.174

*AIS*  Abbreviated Injury Scale, *pRBC*  packed red blood cell(s)

**Fig. 3:**
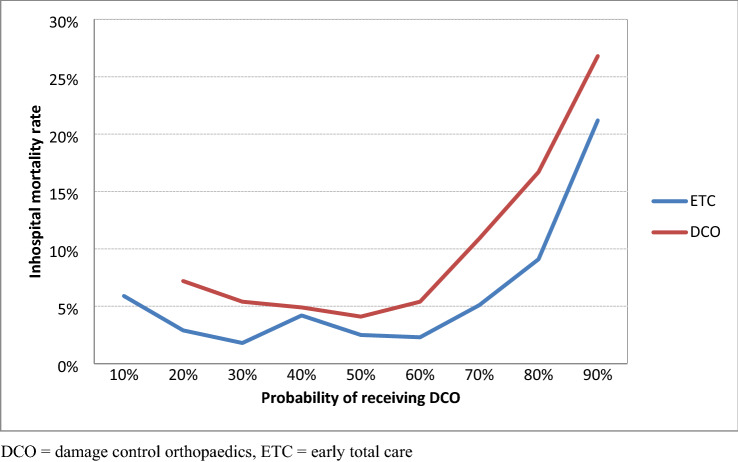
Inhospital mortality for stratified DCO and ETC groups and probability of a patient receiving DCO (propensity scores). *DCO* damage control orthopaedics, *ETC* early total care

Table 5 shows the propensity scores, i.e. the probability of a patient receiving DCO, for different strata. The results confirm that patients with high probability values actually underwent DCO. Regardless of the probability of a patient receiving DCO, as indicated by the propensity score, mortality associated with DCO treatment was, however, always higher than mortality after ETC (Fig. 3).

**Table 5:** Propensity scores for DCO and non-DCO (ETC) patients divided into strata. Stratum 0–9 is not included since there were no data

	ETC	DCO	Total (*n*)
Stratum 10–19	17 (94.4%)	1 (5.6%)	18
Stratum 20–29	210 (71.7%)	83 (28.3%)	293
Stratum 30–39	500 (62.7%)	297 (37.3%)	797
Stratum 40–49	599 (57.2%)	449 (42.8%)	1048
Stratum 50–59	1038 (44.1%)	1316 (55.9%)	2354
Stratum 60–69	897 (34.2%)	1727 (65.8%)	2624
Stratum 70–79	587 (26.3%)	1647 (73.7%)	2234
Stratum 80–89	285 (15.5%)	1556 (84.5%)	1841
Stratum 90–100	52 (6.3%)	72 (93.7%)	824
Total	4185 (34.8%)	7848 (65.2%)	12,033

We also used the propensity score for matching patients who received DCO with patients who underwent ETC treatment. We were able to match 3663 patients in the DCO group (45% of all DCO patients) with 3663 patients in the ETC group (84% of all ETC patients). The results for the matched groups of patients are shown in Table 6. For most variables, similar results were obtained for the two groups. Mortality in the DCO group, however, was more than twice as high as in the ETC group. Complications related to the treatment of a polytrauma patient, such as sepsis, multiple organ failure and pulmonary failure, were also relevantly higher in the DCO group than in the ETC group.

**Table 6:** Clinical findings, scores und outcomes of patients in the DCO group (*n = *3663) and in the ETC group (*n = *3663) who were matched on the propensity score

Parameter	DCO *n = *3663	ETC *n = *3663	*p* value
Age (in years)	46.7 (SD 19.9)	45.7 (SD 20.3)	0.029
Male	68.8% (2521)	70.4% (2580)	0.134
Level 1 (supraregional) trauma centre	86.7% (3176)	85.9% (3148)	0.341
Injury Severity Score	27.9 (SD 10.8)	26.3 (SD 9.9)	< 0.001
Number of injuries	6.9 (SD 3.3)	6.6 (SD 3.0)	< 0.001
Penetrating trauma	1.5% (54)	1.6% (60)	0.571
Head injury (AIS 3 +)	31.9% (1167)	32.5% (1190)	0.565
Thoracic trauma (AIS 3 +)	60.5% (2217)	61.5% (2251)	0.415
Abdominal trauma (AIS 3 +)	20.0% (734)	17.5% (640)	0.005
Injury to an upper extremity (AIS 2 +)	59.2% (2186)	56.4% (2066)	0.016
Injury to a lower extremity (AIS 2 +)	52.7% (1930)	57.6% (2109)	< 0.001
Stable pelvic injury (AIS 2)	15.6% (573)	12.5% (459)	< 0.001
Unstable pelvic injury (AIS 3–5)	27.7% (1016)	28.7% (1053)	
Femur fracture	21.9% (803)	29.1 (1065)	< 0.0.001
Multiple fractures of extremities	48.2% (1765)	39.8% (1459)	< 0.001
Prehospital intubation	40.1% (1469)	36.7% (1343)	0.002
Prehospital fluid administration > 1000 mL	20.1% (736)	25.2% (922)	< 0.001
Coagulopathy	16.2% (595)	14.8% (542)	0.087
Blood transfusions	23.7% (867)	15.6% (572)	< 0.001
Massive transfusions	1.9% (69)	2.2% (80)	0.363
Sepsis	9.2% (314)	6.6% (224)	< 0.0.001
Multiple organ failure	29.3% (1025)	22.8% (792)	< 0.0.001
Pulmonary failure	23.6% (825)	18.1% (628)	< 0.001
Mortality within the first 24 h	2.3% (86)	0.8% (31)	< 0.0.001
Inhospital mortality	8.2% (299)	3.6% (133)	< 0.0.001

*AIS*  Abbreviated Injury Scale, *DCO*  damage control orthopaedics, *ETC*  early total care

## Discussion

The primary objective of this study was to assess the probability that a severely injured patient receives treatment according to the principles of damage control orthopaedics. For this purpose, we calculated propensity scores, which we also used to investigate the effects of DCO, as described by Kuss et al. [29], and to compare the outcomes of damage control orthopaedics and early total care.

In this study, the propensity score allowed us to identify a few variables that make it considerably more probable that the DCO approach is used in the management of patients compared to ETC. Contrary to our expectations, however, our study, which was based on data from the TraumaRegister DGU^®^, did not demonstrate the superiority of DCO over ETC concerning the occurrence of complications such as multi-organ failure, pulmonary failure and sepsis, as well as mortality, although the propensity score is described in the literature as well suited to analysing registry data [29, 31]. Is this problem to prove the advantage of damage control strategies limited to DCO/ETC in polytrauma care? A recent study of DCS in abdominal trauma, also based on data from the Traumaregister DGU^®^ struggled to demonstrate a clear advantage for damage control therapy as well[57]. In another study using data from the Traumaregister DGU^®^, it was possible to show that observed and expected mortality using the RISC score in trauma patients with an AIS max of ≥ 3 in extremity injuries and at least one femur fracture decreased over the years from 2002 to 2018, while the proportion of temporary fixation compared to early total care of the femur increased [5]. In their data analysis, Bläsius et al. found that mainly more severely injured patients with a higher ISS and concomitant injuries received damage control orthopaedics. In addition, this patient group had a higher mortality and a higher rate of multi-organ failure and sepsis. This indicates that the more severely injured patients with an expected worse outcome were more likely to receive DCO therapy, making comparability between ETC and DCO much more difficult. In contrast, in our study we did not look at changes in care over time, but focused on the possible motivations and decisive parameters for using Damage Control Orthopedics. In addition, when using the propensity score in our study, we not only looked at which parameters made a DCO treatment more likely, but also used propensity score matching to make the outcome between ETC and DCO more comparable.

There are two possible reasons for our results. Either DCO is indeed associated with poorer survival than ETC, or adjustment for the variables included here was insufficient to explain why a decision to use or not to use DCO was made. The patient groups showed major differences even in some baseline characteristics (e.g. ISS, shock, massive transfusions) that revealed that patients who received DCO were much sicker than patients in the ETC group. For this reason, we used propensity score matching in order to reduce any possible bias. Even after propensity score matching, the patients in the groups still show differences for example in ISS. These differences are also statistically significant. Whether this difference is relevant due to the very large number of patients is for the reader to decide. Giannoudis et al. reported that the key factors in identifying patients who benefitted the most from DCO were the patients’ overall physiological state and injury characteristics [14]. Following initial care in the resuscitation room, polytrauma patients can be divided into four groups, i.e. stable, borderline, unstable, and in extremis, as proposed by Pape et al. [41]. The more stable the patient is, the more suitable he or she is for ETC. The more unstable the patient is, the more sensible it is to use DCO [41]. However, the presented result in this publication is still only a retrospective study using the propensity score and not a multicenter, prospective randomised trial.

Another possible explanation for the higher mortality and complication rate in our DCO group is that the decision either for or against DCO was made after a relatively long period of time. In severely injured patients who sustained injuries to the chest, abdomen and the extremities, torso injuries are often treated first [19]. During the time from the beginning of care in the resuscitation room and the end of treatment of torso injuries, treatment can have considerable effects on patient stability. These effects are not reflected in the data that are available in the TraumaRegister DGU^®^. In theory, a patient who is initially stable in the resuscitation room and then becomes unstable would be unlikely to receive ETC. DCO would then be associated with a higher probability of mortality because of the patient’s unstable condition. By contrast, a patient who is unstable in the resuscitation room and requires massive transfusions would in general be sufficiently stable to receive ETC after having undergone treatment for torso injuries and damage control resuscitation (DCR). DCR is a group of measures, such as permissive hypotension, early use of blood products, and the limited use of crystalloid fluids, that effectively address the lethal triad of coagulopathy, metabolic acidosis, and hypothermia [12, 32]. In this case, an initially unstable patient would have a lower probability of mortality after ETC. The focus on the patient’s condition in the dynamic initial phase has led to an approach that is referred to as early appropriate care (EAC) [1, 3]. Parameters such as acidosis seem to have emerged that allow early appropriate care after successful damage control resuscitation, despite an initially unstable patient [34]. The proven effectiveness of DCR [42] could also explain why Feldman et al. could not demonstrate an advantage of DCO over ETC in the treatment of femoral shaft fractures [11]. This is because their DCO group consisted of patients from 2007 to 2019, a period in which DCR was increasingly implemented. The ETC group consisted of patients from 1996 to 2006, so it is more than questionable whether a comparison between ETC and DCO is methodological possible at all. That DCR is effective in stabilising unstable patients could also explain the results of the study by Rixen et al., who randomised patients to either DCO or ETC [46]. Rixen et al. reported that the surgeon decided to shift from DCO to ETC or vice versa in three of the 33 patients who had undergone surgery before the study had to be terminated. The surgeon explained that the decisions to perform either DCO (*n = *1) or ETC (*n = *2), which were the result of randomisation prior to surgery, were no longer acceptable. The results of our study suggest that propensity scores work more effectively when treatment decisions and the assessment of variables are closely related in time.

Against this background, the results reported by Yamamoto et al. are particularly surprising [58]. They too used the propensity score and propensity score matching as well in order to compare inhospital mortality after ETC and DCO and reported that DCO was associated with a significantly lower mortality rate. This difference between DCO and ETC was probably attributable to the selection of patients. Yamamoto et al. included all patients who presented with long bone fractures and an AIS ≥ 2 and who underwent definitive care during their hospital stay. They thus excluded all patients who died after initial treatment and before definitive care. This applied to more than 50% of the patients with extremity injuries and thus led to a bias. This approach could also explain why the pulmonary complications in the DCO group were not higher as it is in our study. This is supported by a retrospective study by Santolini et al., who reported that definitive internal fixation after DCO was performed no earlier than after a mean period of 6.7 (± 4.5) days [50]. In our study, 34% of the patients who died in the hospital died within the first 24 h. The proportion of patients who died within the first 24 h is similar to that reported by Bieler et al. in a study on trauma patients [4].

Logistic regression identified a number of parameters that increased the probability of a severely injured patient to receive damage control orthopaedics. These parameters were the transfusion of pRBCs, penetrating trauma, an unstable pelvic fracture, and a lower extremity injury. These parameters are useful predictors of DCO. Many parameters had values close to 1 in the logistic regression. This means that these parameters cannot be clearly allocated to either DCO or ETC. By contrast, polytrauma in children and adolescents and falls from a low height were good predictors of ETC. This is in line with current research on trauma care for severely injured children [26].

The majority of severely injured patients were treated with DCO. Based on the data sets that were analysed in this study, 65.2% of the patients received DCO and 34.8% underwent ETC. This confirms a study that was conducted in 2002 by Pape et al., who reported that the number of polytrauma patients who were treated with DCO continued to increase [38]. When it comes to the management of polytrauma patients in supraregional trauma centres, we found in our study that the number of patients who underwent DCO was almost twice as high as the number of patients who received ETC. In local trauma centres, by contrast, more patients were treated with ETC compared to DCO. Kuhmola et al. reported, however, that only 22% of polytrauma patients were treated with DCO for femoral shaft fractures in a tertiary trauma centre from 2006 to 2018 while 78% underwent ETC [28]. Giannoudis et al. too reported that approximately 20% of polytrauma patients were treated with DCO. They did not state, however, whether this percentage applied to the total group of polytrauma patients or only to polytrauma patients with extremity or pelvic injuries [14]. DCO for the management of polytrauma patients accounted for a percentage of less than 25% in another study as well [9]. Against the background of these percentages, our distribution between the groups studied is nevertheless very surprising. One possible explanation could be that we assigned all patients to the DCO group as soon as only one long bone fracture or a pelvic fracture had been treated with an external fixator in the surgical treatment, even if other fractures had already been definitively treated. After all, almost 60% of the patients in the DCO group had multiple extremity injuries. In the study by Bläsius et al. of the 11409 patients who received surgical treatment, 6241 patients were treated with an external fixator [5]. This means that 54% received a temporary external fixator. This study is also based on the data of Traumaregister DGU^®^, but focuses exclusively on femur fractures, while we look at all long bone and pelvic fractures.

A closer look at our patient group shows that the DCO group included a higher number of patients with signs of shock and consumption coagulopathy, a higher rate of prehospital reanimation, higher pRBC requirements, and a lower Glasgow Coma Scale score. Although most differences between the DCO and the ETC group were not relevant, all investigated clinical parameters suggested that patients in the DCO group at least tended to be sicker and less stable than patients in the ETC group. This finding confirms the results of a study that was published in 2005 by Rixen et al., who investigated the management of femoral shaft fractures in severely injured patients on the basis of TraumaRegister DGU^®^ data [45]. The DCO approach was more commonly applied in patients with a high ISS not only in our study but also in other studies [8, 10, 15, 27, 37, 45]. More severely injured patients are more often treated in supraregional trauma centres and more often undergo DCO. This is confirmed by our results. A comparison of the two treatment approaches during and outside normal hours showed that the percentage of patients who underwent DCO in the on-call setting (67.9% of the DCO patients) was higher than the percentage of patients who received ETC by on-call staff (64.6% of the ETC patients). The difference, however, was not relevant. Dei Guidici et al. [9] too found no relevant difference in the treatment strategies for polytrauma patients who were treated on weekends and those who were admitted on weekdays, although the percentage of DCO patients in their Level 2 trauma centre was much lower than in our study. The objective of DCO is to avoid the burden of the additional surgical trauma associated with ETC, although this implies that a higher number of surgical procedures must be accepted in order to improve patient outcome. There is, however, no clearly defined parameter for outcome. Instead, a variety of outcome parameters can be investigated and defined. In the present study, we analysed the length of stay in the intensive care unit, the mean duration of ventilation (intubation days), the length of hospital stay, the rate of sepsis according to the criteria defined in the TraumaRegister DGU^®^, the rate of patients with multiple organ failure, pulmonary failure, mortality within the first 24 h, and inhospital mortality. Our results revealed some relevant differences between the two patient groups. DCO was associated with a considerably longer ICU length of stay, a considerably higher rate of sepsis, considerably higher rate of pulmonary failure, considerably higher rate of multi-organ failure, considerably higher mortality within the first 24 h, and considerably higher inhospital mortality. Even in our matched groups the rates of sepsis, multi-organ failure, pulmonary failure and mortality were higher in the DCO group. By contrast, Pape et al., who conducted a retrospective cohort study on the treatment of patients with femoral shaft fractures during three different time periods, reported that the increasing use of the DCO approach had led to a decrease in the incidence of multiple organ failure [38]. This finding was confirmed by another study, which also found that DCO treatment was associated with a less severe systemic inflammatory response than ETC [23]. In 2007, Pape et al. published a prospective randomised multi-centre study and reported that even stable patients who were treated with the DCO approach had considerably shorter duration of ventilation and a lower rate of sepsis than patients who underwent primary intramedullary nailing of the femur [40]. Pape et al., however, excluded all critically injured and unstable patients. In a more recent study using data from the Traumaregister DGU^®^, Bläsius et al. found a higher sepsis rate in severely injured patients who had been treated with an external fixator [5].

Many of the studies mentioned in this paper refer only to the treatment of femoral fractures in their comparison between ETC and DCO, respectively between early and delayed definitive fixation [2, 21, 23, 38, 40]. In some cases, these studies only distinguished between primary treatment with an intramedullary nail (ETC) and primary application of an external fixator (DCO). However, if the aim of the damage control techniques is also to minimise the second hit through initial surgical treatment [17, 18, 24], these studies show only part of the truth, as they do not take into account the treatment of all other limb fractures and the treatment by other ETC techniques. In our study fractures of the pelvis as well as all fractures of long bones of the extremities were included. This could also be a possible explanation why, in contrast to our study, these studies often showed a significant reduction in complications such as ARDS and multi-organ failure when using DCO, because the use of an intramedullary nail for femoral fractures is relatively often associated with pulmonary complications [25, 56]. On the other hand, there is a consensus among experts that early stabilisation of long bone fractures is widely accepted among experts in the treatment of severely injured patients, while other DCO indications have been dropped [44].

The way the DCO and ETC groups were formed in our study has its limitations, which must be kept in mind when our results are discussed. For example, the DCO group included patients who had been treated with external fixation of pelvic and/or long bone fractures. We cannot be sure that external fixation was always used as a DCO technique. It is possible that external fixation was used as a definitive fracture fixation procedure. In addition, documentation may have been incomplete or incorrect so that patients may have been allocated to the wrong group. This may have affected the accuracy of differentiation between the study groups. In addition, it is possible that the patient in the DCO group received a mixed treatment partly by means of ETC and DCO, as we assigned the patients to the DCO group as soon as at least one extremity and/or pelvic fracture was treated with an external fixator. But stabilising of all long bone fractures with an external fixator can be an overuse of external fixators, so the strategy of safe definitive surgery (SDS) was established, which is a dynamic synthesis of ETC and DCO depending on clinical parameters and repeated reassessments [35, 55]. Against this background, it has to be discussed whether the DCO group of our study is not actually an SDS group according to Pape and Pfeiffer [43], since in many cases both temporary fracture stabilisation and early total care were performed in combination and our study included patients from the years 2009 to 2015, and thus DCR with the repeated reassessments was certainly already established. The way we formed our groups could thus also explain why about 65% of our patients were assigned to the DCO group, even though Bläsius et al. also found a significant increase in the use of the DCO concept [5]. In their study 47.1% were treated with an external fixator. As described earlier in the chapter, the proportion of DCO increases in this study to 54.3% when conservatively treated patients are excluded as in our study.

Another limitation of our study is that we only included patients with pelvic fractures and fractures of the long bones. Patients with e.g. spinal injuries without pelvic and/or extremity injuries were not considered, although damage control procedures are described here as well [16].

The time between the patient's arrival and the treatment was not taken into account. However, this is in line with the changed approach that not all fractures have to be stabilised within 24 h after the trauma [42], even though it should be done if possible [44].

For these reasons, our study cannot provide definitive evidence for the superiority of ETC over DCO although the results appear to do so at first glance. Furthermore, terms such as ETC, DCO, EAC and SDS do not seem to be one hundred percent sharply defined. They are rather terms that are used to try to describe certain surgical and tactical strategies. Hafner et al. as well as Rondanelli et al. come to the conclusion that after the establishment of ETC and DCO as well as in the further course of EAC and SDS, patients in an initially unclear condition (borderline patients) now also can be treated as quickly as possible with an individually adapted treatment concept [20, 48].

## Conclusions

There is clear evidence supporting the damage control orthopaedics approach in the management of patients. Based on the propensity score, however, our study could not show that DCO, defined in this study as early temporary fracture stabilisation with an external fixator of at least one fracture of the pelvis and/or extremity long bone fracture was superior to ETC, which we defined as early definitive fracture fixation without prior temporary stabilisation concerning typical complications such as multi-organ failure, pulmonary failure and sepsis as well as the mortality in severely injured patients. Certain parameters of polytrauma patients used in the trauma registry (Traumaregister DGU^®^) are more frequently associated with the use of DCO therapy.

The use of terms such as DCO and ETC helps on the one hand to describe different strategies. In everyday clinical practice, however, the different terms and strategies mentioned in this study are sometimes merged with each other in such a way that a very clear separation hardly seems possible for a retrospective analysis of registry data.

## Abbreviations

AIS: Abbreviated Injury Scale
ARDS: Adult respiratory distress syndrome
BP: Blood pressure
DCO: Damage control orthopaedics
DCR: Damage control resuscitation
DGU: German Trauma Society
ETC: Early total care
INR: International Normalised Ratio
GCS: Glasgow Coma Scale
ICU: Intensive care unit
IQR: Interquartile range
ISS: Injury Severity Score
pRBC: Packed red blood cell(s)
OR: Odds ratio
PTT: Partial thromboplastin time
SD: Standard deviation
